# Duration of the susceptibility of pruning wounds of different ages to infections by *Phaeomoniella chlamydospora* on grapevine cv. Cabernet Sauvignon in Central Chile

**DOI:** 10.3389/ffunb.2022.1026516

**Published:** 2022-11-28

**Authors:** Gonzalo A. Díaz, Bernardo A. Latorre

**Affiliations:** ^1^ Laboratorio de Patología Frutal, Departamento de Producción Agrícola, Facultad de Ciencias Agrarias, Universidad de Talca, Talca, Chile; ^2^ Departamento de Fruticultura, Facultad de Agronomía e Ingeniería Forestal, Pontificia Universidad Católica de Chile, Santiago, Chile

**Keywords:** fungal trunk pathogen, chilean vineyards, grapevine trunk diseases (GTDs), pruning wound infection, age of pruning wound

## Abstract

Grapevine trunk diseases (GTDs) are one of the most important phytosanitary problems that affect grapevines (*Vitis vinifera*) worldwide. In Chile, *Phaeomoniella chlamydospora* is the major fungal trunk pathogen associated with GTDs. In the vineyards, the natural infections by *P. chlamydospora* are associated with air-borne conidia dispersed onto fresh pruning wounds from pycnidia. These pruning wounds are considered an important entrance for fungal trunk pathogens such as *P. chlamydospora* in the host in the field. However, the duration of the susceptibility of grapevine annual pruning wounds to *P. chlamydospora* is still unknown in Chile. Therefore, this study aimed to evaluate the period of susceptibility of pruning wounds of different ages to artificial infection of *P. chlamydospora* on grapevine cv. Cabernet Sauvignon, Central Chile. Artificial inoculations of a conidial suspension (10^5^ conidia/mL) of *P. chlamydospora* were used to determine the susceptibility of pruning wounds of different ages, from 1, 15, 30, and 45 days after pruning. The experiments were conducted on lignified cuttings in a greenhouse, and on vine spurs in two vineyards (Buin and Nancagua, Central Chile) during two consecutive seasons. The results indicated that the pruning wounds of grapevine cv. Cabernet Sauvignon were very susceptible to infections by *P. chlamydospora*, with a percentage of pruning wounds infected from 97 to 71% for cuttings, and 96% to 60% for spurs, during the first 15 days after pruning. However, the susceptibility of pruning wounds of different ages in cuttings and spurs of grapevine, generally decreased as the time from pruning to inoculation increased. Moreover, the pruning wounds the pruning wounds remained susceptible to artificial inoculation by *P. chlamydospora* for up 45 days after pruning with percent of wounds infected from 8.0 to 12.2, and 8.3 to 18.8% on cuttings and spurs of grapevine, respectively. Finally, this study constitutes study constitutes the first research focalized on the susceptibility of pruning wounds of various ages of grapevine cv. Cabernet Sauvignon to artificial inoculations by *P. chlamydospora* in Central Chile.

## Introduction

Grapevine (*Vitis vinifera* L.) is a crucial fruit crop that is cultivated in several countries in both hemispheres (Food and Agriculture Organization of the United Nations, 2022; www.fao.org/faostat). Chile is one of the leading exporters of wines with over 136,166 ha dedicated to wine production, which are concentrated in the central zone between the Metropolitana (33°42’S; 70°39’W) and Maule regions (35°25’S; 71°40’W). The export of wine reached 908 million liters during the 2021 growing season, representing an important agricultural activity for the economy in terms of productivity and employment creation, and accounting for USD$ 2,037 million (Odepa, 2022; www.odepa.cl). The main cultivars planted were Cabernet Sauvignon and Sauvignon Blanc, representing 29.4% and 11.2% of total grapevines, respectively (Odepa, 2022; www.odepa.cl). Central Chile is characterized by a Mediterranean climate (Csb) with a warm and dry summer and cold, wet winters with main annual rainfall between 342 and 676 mm (Sarricolea et al., 2017). Under these weather conditions, Cabernet Sauvignon is the most planted cultivar, mainly under irrigation and trained as a bi-lateral cordon trellis (Gil and Pszczólkowski, 2015).

Grapevine trunk diseases (GTDs) such as Botryosphaeria dieback, Eutypa dieback, Petri disease, Esca disease, and Esca-like disease, which are caused by fungal pathogens, are one of the most important phytosanitary problems that affect *V. vinifera* in Chile (Díaz et al., 2013; Larach et al., 2020; Lolas et al., 2021) and worldwide (Mugnai et al., 1999; Cloete et al., 2015; Gramaje et al., 2018; Kenfaoui et al., 2022). GTDs cause a gradual dieback of arms and trunks, reducing plant longevity, the overall yields, quality, and eventually the death of the entire plant (Úrbez-Torres et al., 2006; Úrbez-Torres et al., 2014; Gramaje et al., 2018; Larach et al., 2020). Several fungal trunk pathogens have been isolated and described from symptomatic grapevines with GTDs, including *Phaeomoniella chlamydospora* (W. Gams, Crous, M. J. Wingf. & Mugnai) Crous & W. Gams, *Phaeoacremonium* spp., and species belonging to families of Botryosphaeriaceae as *Diplodia seriata* De Not. and *Neofusicoccum parvum* (Pennycook & Samuels) Crous, Slippers & A.J.L. Phillips., and Diatrypaceae such as *Eutypa lata* (Pers.) Tul. & C. Tul., and *Cryptovalsa ampelina* (Nitschke) Fuckel., among others (Mugnai et al., 1999; Aroca et al., 2010; Gramaje et al., 2018; Kenfaoui et al., 2022).

In Chile, *P. chlamydospora* is one of the main fungal trunk pathogens associated with Petri and Esca-like diseases. It is consistently involved with black-wood streaking symptoms and characterized by the presence of dark-brown to black discoloration of xylem tissue in the wood (Díaz et al., 2013; Díaz and Latorre, 2014). However, other fungal trunk pathogens, such as the species Botryosphaeriaceae and Diatrypaceae have also been reported in Chile and are associated with GTDs (Díaz et al., 2011; Morales et al., 2012; Díaz et al., 2013; Díaz and Latorre, 2014; Lolas et al., 2020; Gaínza-Cortés et al., 2020).

**Figure 1:**
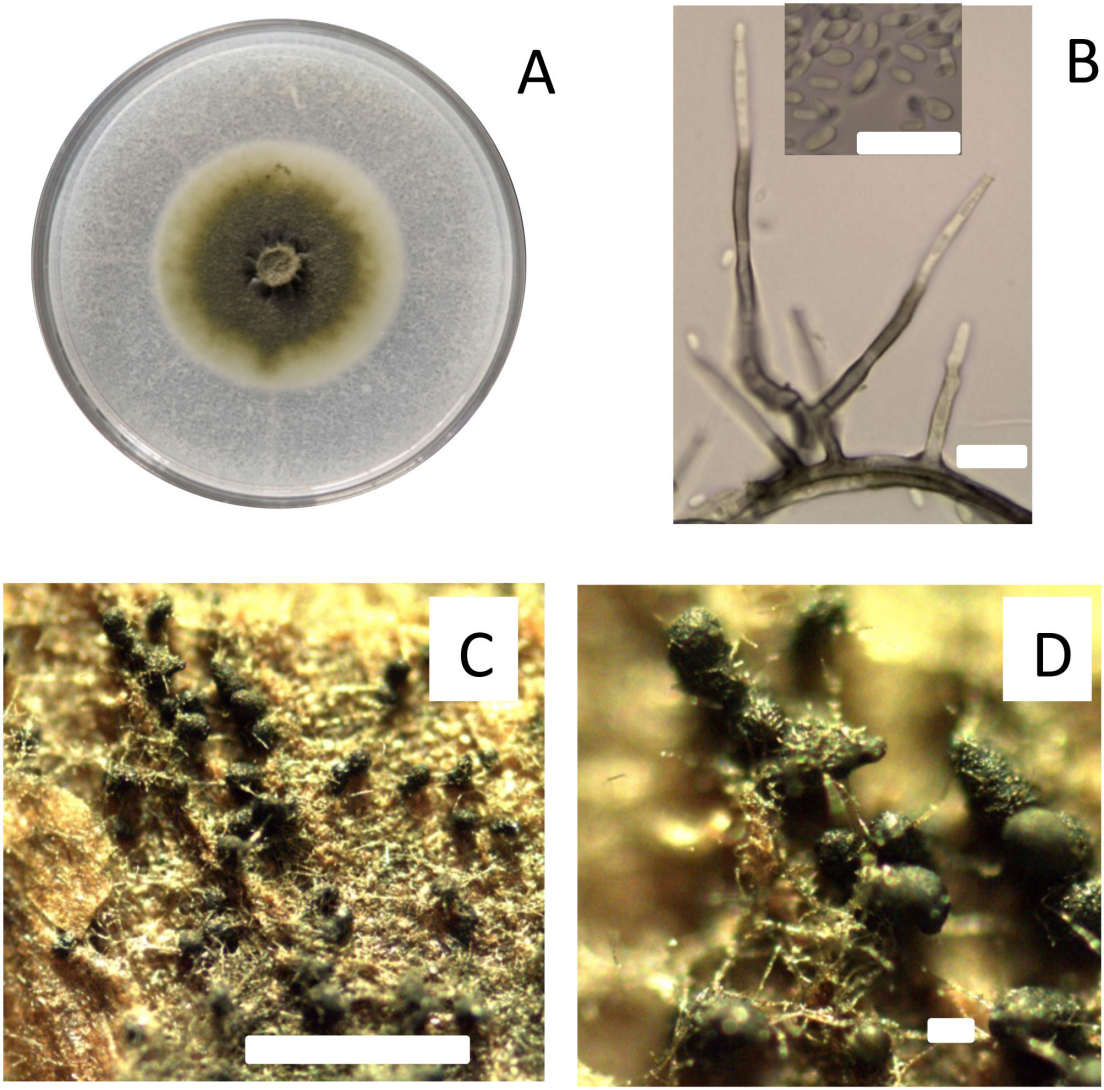
Characteristics of isolate Pach 3 of *Phaeomoniella chlamydospora* studied as fungal trunk pathogen on the susceptibility of pruning wound of different ages of Cabernet Sauvignon in central Chile. Colony of *P. chlamydospora* after 21 days incubated at 25°C on PDA media **(A)**. Morphological characteristics of *P. chlamydospora* as conidiophores and conidia **(B)**. Inoculum of pycnidia of *P. chlamydospora* induced on autoclaved grapevine wood chips on WA media **(C, D)**. All bars = 10 um, except C and D with bars of 200 um and 1000 um, respectively.

The fungus *P. chlamydospora* is a trunk pathogen that is characterized by slow mycelial growth *in vitro*, initially producing a yeast-like colony of whitish and shiny, turned to dark olivaceous with sparse aerial mycelium with age (Mugnai et al., 1999; Crous and Gams, 2000; Pascoe and Cottral, 2000). Conidiophores are erect, branched, smooth, and septated, where the base is subcylindrical, green-brown, but hyaline toward the tip (**Figure 1**). The conidia are unicellular, slightly pigmented, smooth, oblong to ellipsoidal, produced in aggregates from the conidiophore apex (Crous and Gams, 2000; Pascoe and Cottral, 2000; Díaz and Latorre, 2014). In laboratory conditions, conidia of *P. chlamydospora* are produced by conidiophores and phialide from aerial mycelia and pycnidia on cultures (pine needle or chip woods of grapevine), and pycnidia developed on the surface of plantlets, shoots, and vines inoculated with *P. chlamydospora* (Mugnai et al., 1999; Pascoe and Cottral, 2000; Díaz and Latorre, 2014). Furthermore, various studies conducted in vineyards in Australia, California, and South Africa have found the pycnidia state of *P. chlamydospora* on the surface of pruning debris (ground) and diseased tissues (cordons and old spurs) in the vine (Edwards and Pascoe, 2001; Eskalen et al., 2002; Baloyi et al., 2016). Although Edwards and Pascoe (2001) found pycnidia of *P. chlamydospora* in vineyards, the viability of conidia was insignificant and considered as spermatia. However, studies by Eskalen et al. (2002; 2004) and Baloyi et al. (2016) in vineyards in California and South Africa demonstrated that conidia obtained from pycnidia on the surface of diseased tissues were viable and pathogenic. Therefore, these diseased tissues, including pruning debris left in the ground, cordons, and old spurs attached to the grapevine, are considered important inoculum sources in commercial vineyards (Baloyi et al., 2016; Gramaje et al., 2018). However, knowledge about the epidemiology of Petri and Esca diseases and the role of *P. chlamydospora* is still limited.

Natural infection has been associated with air-borne conidia of fungal trunk pathogens, which are dispersed on fresh pruning wounds during rainy periods during winter months (Eskalen and Gubler, 2001; Eskalen et al., 2002; van Niekerk et al., 2010; Úrbez-Torres et al., 2010). In this sense, conidia of *P. chlamydospora* have been detected and quantified in the field, being correlational with the occurrence of rainfall or high relative humidity, especially during winter months in California (Eskalen et al., 2004) and South Africa (van Niekerk et al., 2010). Natural infections by *P. chlamydospora* on pruning wounds were detected in a diseased vineyard (30 years old) and healthy vineyards (10 years old), with 21 and 0% of grapevines showing GTDs symptoms, respectively, in Catalonia, Spain (Luque et al., 2014). Therefore, it has been hypothesized that the major entrance for fungal trunk pathogens in the host is the pruning wound. Several inoculation studies have revealed that *P. chlamydospora* can cause black wood streaking from pruning wound infections (Eskalen et al., 2007; Serra et al., 2008; Elena and Luque, 2016). Previous studies performed in vineyards with inoculation of fungal trunk pathogens, including *Eutypa lata*, Botryosphaeriaceae, and *P. chlamydospora* on pruning wounds, have shown that the susceptibility was highest to infection when the inoculation was done on fresh pruning wounds (the first two weeks), but the susceptibility decreased on the wounds when the time between pruning wounds and inoculation increased (van Niekerk et al., 2011; Úrbez-Torres and Gubler, 2011; Elena and Luque, 2016). Moreover, according to studies on the grapevine, the pruning wounds inoculated with *P. chlamydospora* can be susceptible for a period extended up to 4 to 16 weeks after pruning from vineyards in California (Eskalen et al., 2007), France (Chapuis et al., 1998), Italy (Serra et al., 2008), South Africa (van Niekerk et al., 2011) and Spain (Elena and Luque, 2016). For the development of a strategy in the management of the GTDs, studies about the duration of pruning wounds are important to elucidate aspects such as time for protection with fungicides and biological agents according to the susceptibility of pruning wounds. However, the duration of the susceptibility of grapevine pruning wounds is still unknown in Chile. Therefore, this study aimed to evaluate the period of susceptibility of pruning wounds of different ages to artificial infection of *P. chlamydospora* on grapevine cv. Cabernet Sauvignon in Central Chile.

## Material and methods

### Fungal isolate and inoculum

This study used *P. chlamydospora* isolate Pach-3, obtained from vascular discoloration developed in grapevines showing Esca-like symptoms. This isolate was identified morphologically and molecularly (Díaz and Latorre, 2014) (**Figure 1**), and was kept in the fungal collection at the Plant Pathology Laboratory, Faculty of Agricultural Science, University of Talca, Chile. The isolate Pach-3 was recovered and maintained on 2% potato dextrose agar (PDA) at 25°C for further study (**Figure 1**).

The inoculum consisted of a conidial suspension obtained from pycnidia induced on autoclaved grapevine wood chips (1 cm^2^) that were aseptically placed onto 2% water agar (WA). Mycelium plug (5-mm diameter) of 15 days-old culture of Pach-3 was placed in both extremes of wood chips on WA and incubated for 21 days at 25°C under near UV light with a regime of 12 h of photoperiod, and then another 21 days incubated at 10°C (Díaz and Latorre, 2014) (**Figure 1**). Pycnidia were collected and crushed in 1 mL of sterile distilled water with 0.05% Tween 80 (polysorbate surfactant; Sigma-Aldrich; Missouri, USA) to release the conidia. The conidial suspensions used were adjusted to a concentration of 10^5^ conidia/mL of *P. chlamydospora* using a hemocytometer (Serra et al., 2008; Díaz and Latorre, 2014) and stored at 4°C until inoculation to avoid early spore germination. The conidial viability of each inoculum suspension was tested after incubating 100 uL of each conidial suspension on 2% WA at 25°C (Úrbez-Torres and Gubler, 2011). Percent germination was determined after 12 h of incubation, and at least 100 conidia were observed. A spore was considered germinated if the length of the germ tube was at least twice the length of the spore.

### Effect of the age of pruning wounds on infection of *P. chlamydospora* in grapevine cuttings

Healthy lignified dormant canes (50 cm long) of one-year-old of grapevine (*V. vinifera*) cv. Cabernet Sauvignon were collected during the second week of June (dormant seasons 2014 and 2015) from an apparently healthy commercial vineyard located in Buin (33°43´S; 70°41´W), Central Chile. Cuttings were transported to the laboratory and maintained at 5°C. The dormant cuttings were washed with sterile distilled water, and then 10 dormant cuttings were placed at a 90° angle at 10 cm in depth in a polyethylene box (35 x 30 x 15 cm) containing humid perlite (75% relative humidity, RH).

The tips of the cuttings were pruned off at a 45° angle with the aid of a manual disinfested pruning shear. Wounds were inoculated at 1, 15, 30, and 45 days after pruning with a 40 uL drop of a conidial suspension placed with a micropipette on top of each wound (Serra et al., 2008). An equal number of grapevine cuttings treated with 40 uL of sterile distilled water were left as negative controls. All cuttings were incubated for 4 months in a greenhouse (18-25°C, 70-80% RH), before determining the length of the vascular discoloration (mm) developed downwards from the pruning wounds using an electronic caliper.

To determine the proportion of infected pruning wounds, small fragments (5 mm) of necrotic tissues from inoculated cuttings were surface disinfected, sprayed with ethanol (75°), dried under a flow hood, and placed on Petri dishes containing modified PDA (2%) with 0.005% tetracycline, 0.01% streptomycin, and 0.1% Igepal CO-630 (Sigma-Aldrich) (Díaz and Latorre, 2014). Plates were incubated at 25°C with a 12 h photoperiod. The pathogen reisolated was identified by colony characteristics, growth rate, and conidia morphology (Crous and Gams, 2000; Díaz and Latorre, 2014). This experiment was repeated two times.

### Effect of the age of the pruning on infection of *P. chlamydospora* in the vineyard

Field studies were performed in the 2014 and 2015 dormant seasons in two irrigated commercial vineyards of 12- and 15-year -old cv. Cabernet Sauvignon located in Maipo Valley and Cachapoal Valley, in Central Chile, respectively. Vines were trained as bi-lateral cordons and spur prune during the experiments. Both vineyards were considered of low prevalence, with values estimated between 3 and 7% of grapevines with symptoms of Esca-like disease (Díaz and Latorre, 2014). The detection of *P. chlamydospora* was positive in 0.4 and 2.1% of grapevine showing symptoms of Esca-like in vineyards located in Maipo and Cachapoal, respectively.

One-year-old canes were spur-pruned to three buds during dormancy in the last week of June. The pruning wounds were made with disinfested pruning shears at an angle of 45° and 2-3 cm above the third bud in each spur (Elena and Luque, 2016). Based on Petzoldt et al. (1981), 2 h before inoculation, all pruning wounds were slightly wetted by spraying 3 mL of sterile distilled water, in order to simulate rain and to assure an even distribution of the spores over the wound surface. Pruning wounds were directly inoculated at various ages of pruning wounds, with a 40 uL drop of a conidial suspension (10^5^ conidia/mL) placed with a micropipette on each pruning wound (Serra et al., 2008). Pruning wounds were inoculated at 1, 15, 30, and 45 days after pruning (age of pruning wound). Control pruned canes were treated with 40 uL of sterile distilled water. Eight months after, the spurs were excised about 15 cm below the point of inoculations and brought to the laboratory for measurement of the extent of vascular discoloration (mm). The percentage of infected wounds (reisolation, %) was determined by taking small fragments (5 mm) from the margins of necrotic tissues of inoculated spurs, disinfecting them, and placing them on Petri dished with PDA modified (Díaz and Latorre, 2014). They were then incubated at 25°C. The pathogen reisolated was identified by colony characteristics, rate of growth, and spore morphology (Crous and Gams, 2000; Díaz and Latorre, 2014).

### Data analysis

Control treatments in both experiments were excluded from statistical data analysis. This information was only used to estimate the potential natural infection from the natural inoculum in each vineyard.

The experiments of dormant cuttings were arranged using a completely randomized design. Vascular discoloration (mm) data and percent of infection (%) were analyzed separately as a two x four factorial (years x age of pruning wounds), with four replicates and ten dormant canes as the experimental units. Percent of wounds infected (%) data were transformed using the arcsine of the square root of the proportion prior to analyses. Data were studied for an analysis of variance (ANOVA), and means were subjected to a pairwise multiple comparison test of Tukey (*P* < 0.05) using SigmaStat 12.0 (Systat Software Inc., San José, CA, USA).

In the vineyard experiments, the inoculation treatments were arranged as a randomized complete block design, where each year was analyzed separately. Vascular discoloration (mm) and percent of wounds infected (%) data were analyzed independently, as a two x four factorial (locations x age of pruning wounds), with eight replicates and eight spurs in two consecutive vines as the experimental units. Percent of wounds infected (%) data were transformed using the arcsine of the square root of the proportion before analysis. Data were studied for an analysis of variance (ANOVA), and means were subjected to a pairwise multiple comparison test of Tukey (*P* < 0.05) using SigmaStat 12.0 (Systat Software Inc., San José, CA, USA).

### Weather data

The daily average temperature and accumulated rainfall data were obtained from an automatic weather station placed in Buin and Nancagua, about 200 m from both vineyards. Weather data for the whole study were obtained from the weather service of the vineyards during two consecutive seasons (January to December 2014 and 2015).

## Results

### Effect of the age of pruning wounds on infection by *P. chlamydospora* in grapevine cuttings

**Figure 2:**
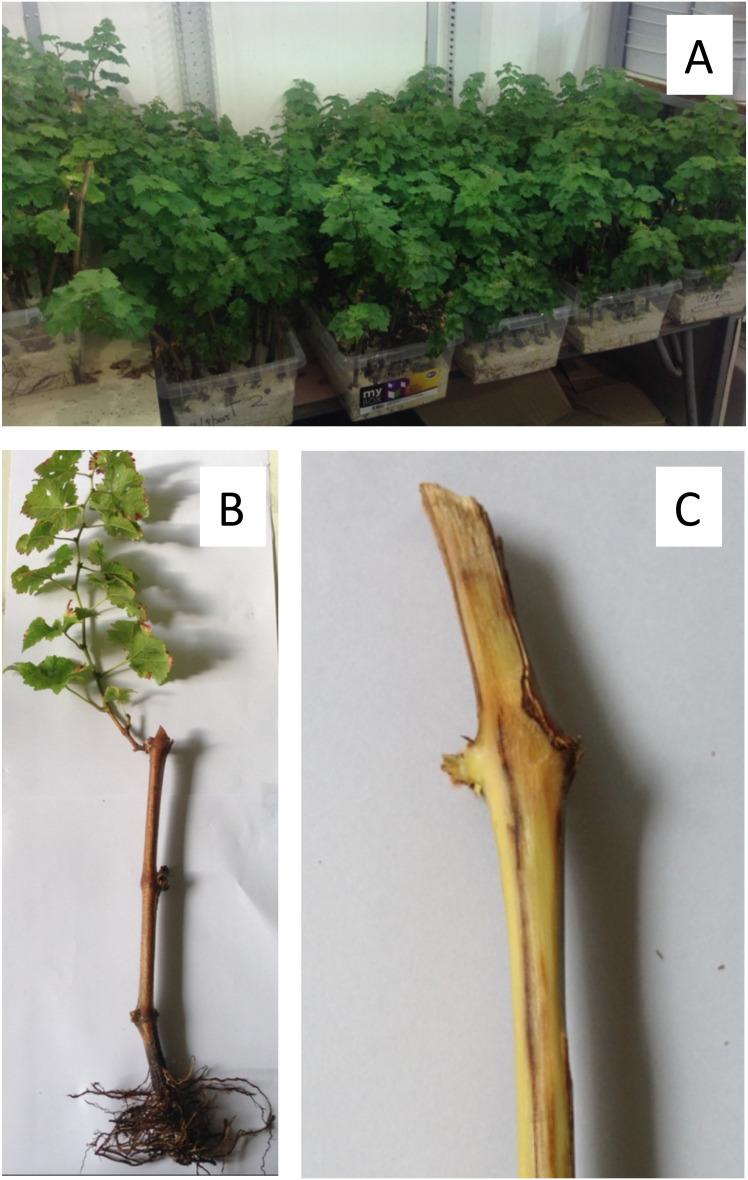
Rooted cuttings of one-year-old of grapevine cv. Cabernet Sauvignon inoculated with a conidial suspension of isolate Pach-3 of *Phaeomoniella chlamydospora* under greenhouse conditions (with temperature between 15-24°C and 70-85% HR) for four months. General view of part of grapevine cuttings after four months **(A)**. Apparently healthy grapevine cuttings inoculated with *P. chlamydospora* showed green shoots and root system **(B)**. Grapevine cutting shows vascular discoloration, which was inoculated one day after pruning **(C)**.

All grapevine cuttings were viable and developed roots and shoots under greenhouse conditions (**Figures 2A, B**). Cuttings developed vascular discoloration (**Figure 2C**) after pruning wound inoculations with a conidial suspension of *P. chlamydospora* (Pach-3). The analysis of the variance of vascular discoloration data showed no significant year x age of pruning wound interaction (*P*=0.06; *F*=2.82). The extent of vascular discoloration was significant according to the age of the pruning wound (**Table 1**), where the length of vascular discolorations significantly decreased from 62.1 mm to 8.6 mm after 1 day to 45 days of pruning, respectively (**Table 1**). The year of the experiment of cutting inoculated showed significant vascular discoloration (*P*=0.006; *F*=9.08), with an extent of vascular discolorations of 31.6 and 25.9 mm for 2014 and 2015, respectively (**Table 1**).

**Table 1:** Vascular discoloration (mm) caused by artificial inoculations of *Phaeomoniella chlamydospora* (Pach-3) on pruning wound of rooted cutting (one-year-old) inoculated at different times after pruning (1, 15, 30, and 45 days), under greenhouse conditions (with temperature between 15-24°C and 70-85% HR) during four months.

Age of pruning wound (days)	Vascular discoloration (mm)			Percent of grapevine pruning wounds infected (%)		
	2014	2015	Mean^a^	2014	2015	Mean^a^
1	66.0	58.1	62.1 d	93.8	100	96.9 b
15	33.7	20.4	27.1 c	75.0	68.8	71.9 b
30	19.0	15.6	17.3 b	25.0	37.5	31.3 a
45	7.7	9.4	8.6 a	12.5	25.0	18.8 a
Mean^1^	31.6 B	25.9 A		51.6	57.8	
*ANOVA*	df	*P*	SED^b^	df	*P*	SED^b^
Year (Y)	1	0.006	3.9	1	0.838	4.8
Age (A)	3	< 0.001	2.7	3	< 0.001	2.2
Y x A	3	0.060	6.9	3	0.847	7.1

^a^Means of vascular discoloration (mm) and percent of wounds infected (%) followed by the different upper case letter in each row or lower case letter in each column differ significantly according to Tukey´s pairwise multiple comparison test. ^b^SED: Standard error of the difference.

The effect of the age of pruning wounds on the percent of wounds infected was significant (*P*=0.001; *F*=26.97), with the mean percentage of wounds infected decreasing from 96.9 to 18.8%. However, the year factor (*P*=0.83; *F*=0.04) and the interaction between year x age of pruning wounds (*P*=0.841; *F*=0.27) had no significant effect on the percentage of wounds infected (**Table 1**).

**Figure 3:**
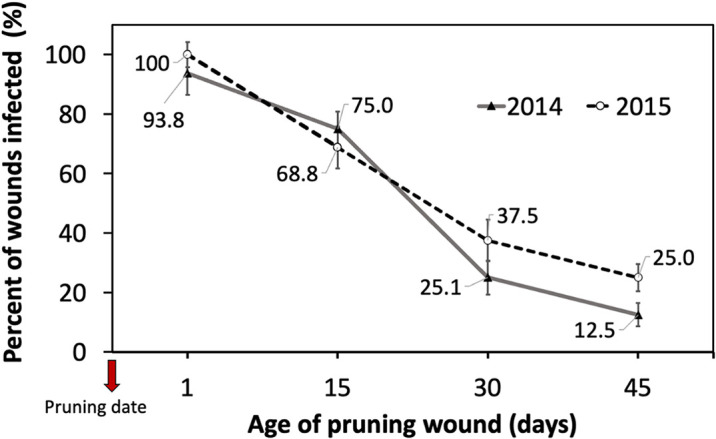
Percent of grapevine pruning wounds infected expressed as mean (%) ± standard deviation of rooted cutting of one-year-old cv. Cabernet Sauvignon by artificial inoculation of 40 uL of a conidial suspension (10^5^ conidia/mL) of *Phaeomoniella chlamydospora* on pruning wounds of different ages (1, 15, 30, and 45 days after pruning) under greenhouse conditions (with temperature between 15-24°C and 70-85% HR) during four months in dormant seasons 2014 (line) and 2015 (segmented line).

The cutting susceptibility estimated by the percentage of wounds infected generally declined when the age of the pruning wound increased during the two years of experiments (**Figure 3**). Differences in the mean percent of wounds infected between inoculations were measured 1 and 15 days after pruning, and inoculations performed 30 and 45 days after pruning were significant (**Table 1**).

### Effect of age of pruning wound of grapevine spurs on infections by *P. chlamydospora* in vineyards

**Figure 4:**
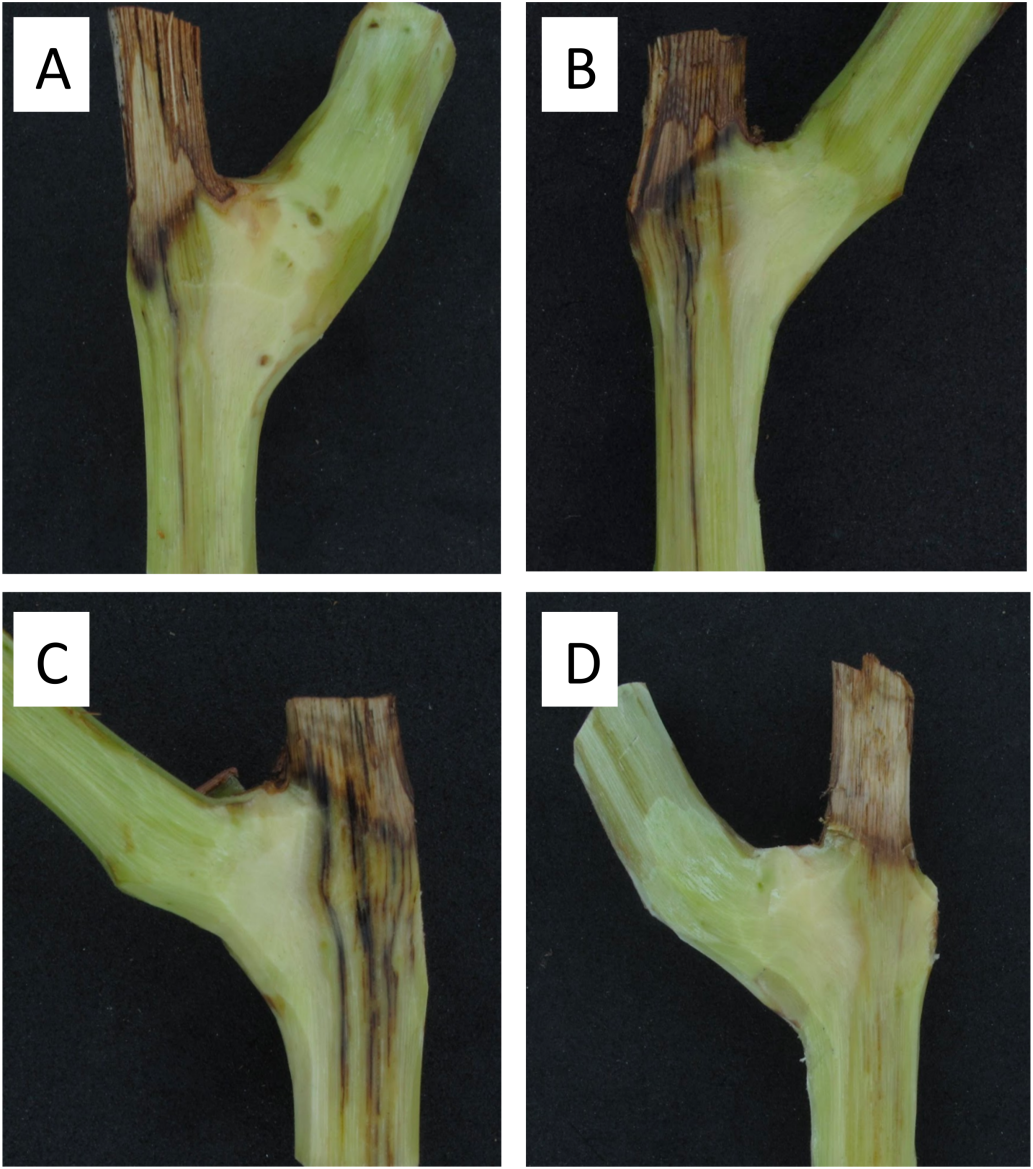
Vascular discoloration caused by artificial inoculation of a conidial suspension (10^5^ conidia/mL) of *Phaeomoniella chlamydospora* (Pach-3) on pruning wounds of different ages of spurs cv. Cabernet Sauvignon after eight months in Central Chile. Vascular discoloration on spur inoculated 15 days after pruning in a vineyard located in Buin **(A)**. Necrotic lesion on spur inoculated 1 day after pruning in a vineyard located in Buin **(B)**. Necrotic lesion on spur inoculated 1 day after pruning in a vineyard located in Nancagua **(C)**. Healthy spur of non-inoculated pruning wound in natural conditions of commercial vineyard located in Nancagua **(D)**.

All pruning wounds on artificially inoculated spurs developed vascular discolorations after 8 months in the vineyards localized in Buin and Nancagua in 2014 and 2015 (**Figure 4**). The mean length of vascular discoloration varied from 34.9 to 41.6 mm for the experiments conducted in Buin and Nancagua, respectively, in 2014 and from 23.8 to 29.1 mm for Buin and Nancagua, in 2015 respectively (**Table 2**). No natural infection of *P. chlamydospora* was detected in both vineyards from the control treatment spurs during the 2014 and 2015 growing seasons.

**Table 2:** Vascular discoloration (mm) caused by artificial inoculation of *Phaeomoniella chlamydospora* at different ages of pruning wounds (1, 15, 30, and 45 days after pruning) in the commercial vineyards cv. Cabernet Sauvignon during eight months in two localities (Buin and Nancagua), Central Chile.

Age of pruning wound (days)	Vascular discoloration (mm)					
	Locality, 2014^a^			Locality, 2015^a^		
	Buin	Nancagua	Mean	Buin	Nancagua	Mean
1	57.5 d	77.9 e	67.7	49.1 e	58.2 e	53.7
15	47.2 d	48.3 d	47.8	19.7 c	34.4 d	27.1
30	22.6 bc	32.4 c	27.5	18.4 bc	14.9 abc	16.7
45	12.2 ab	8.0 a	10.1	8.1 a	9.0 ab	8.6
Mean	34.9	41.6		23.8	29.1	
*ANOVA*	df	*P*	SED^b^	df	*P*	SED^b^
Age (A)	3	< 0.001	1.9	3	< 0.001	1.5
Locality (L)	1	< 0.001	1.3	1	0.002	1.1
A x L	3	< 0.001	2.7	3	< 0.001	2.2

^a^Means of vascular streaking (mm) followed by the different lower case letters in each row and column differ significantly according to Tukey´s pairwise multiple comparison test. ^b^SED: Standard error of the difference.

Independent of the year studied, the length of the vascular discoloration that developed on the spurs was significantly affected by the locality and the age of the pruning wound (**Table 2**). The interaction age x locality was also significant (*P*<0.001; *F*=7.63) (**Table 2**).

**Figure 5:**
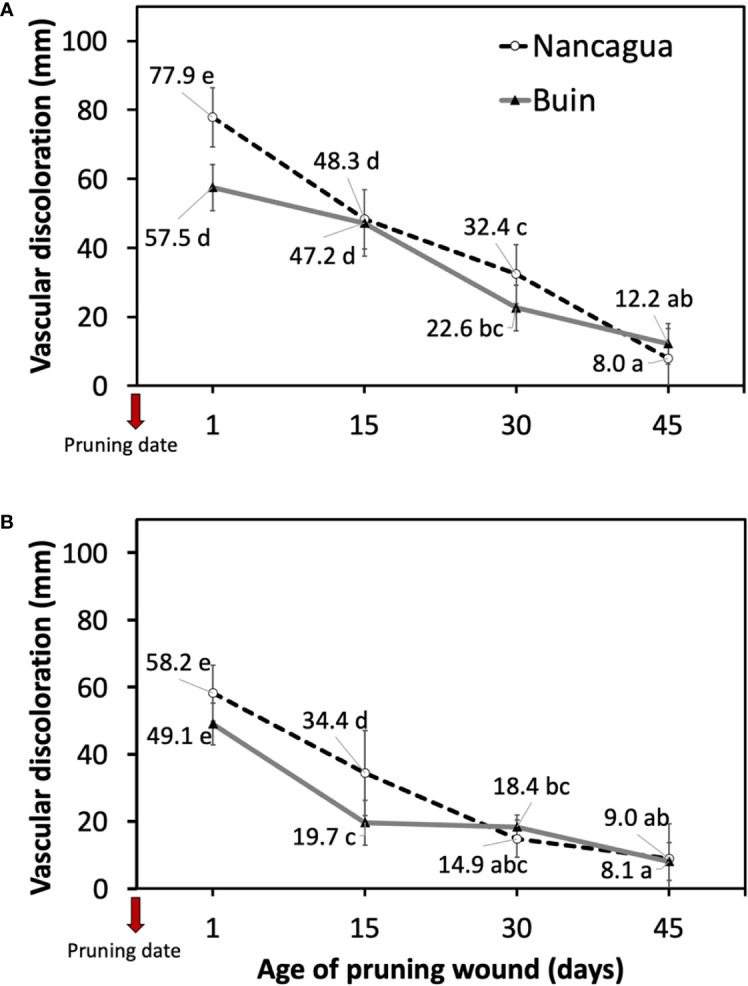
Lesions of vascular discoloration expressed as mean (mm) ± standard deviation caused by artificial inoculation of 40 uL of a conidial suspension (10^5^ conidia/mL) of *Phaeomoniella chlamydospora* on pruning wound of different ages of spurs cv. Cabernet Sauvignon at 1, 15, 30, and 45 days after pruning in the commercial vineyards located in Buin (line) and Nancagua (segmented line), during eight months from the dormant season of 2014 **(A)** and 2015 **(B)** in Central Chile.

In 2014, the *P. chlamydospora* inoculated at 1 and 15 days after pruning developed a vascular discoloration of 77.9 mm (Nancagua) and 47.2 mm (Buin), with significantly more extensive lesions than the other ages of pruning wounds (**Table 2**; **Figure 5**). In 2015, inoculation of *P. chlamydospora* on fresh pruning wounds (one day of age of pruning wounds) produced significantly more extensive vascular discoloration, with values of 58.2 mm (Nancagua) and 49.1 mm (Buin), than the vascular discoloration obtained from spurs inoculated at 15, 30, and 45 days after pruning, with values of 34.4 mm (Nancagua) to 8.1 mm (Buin) (**Figure 5**).

**Figure 6:**
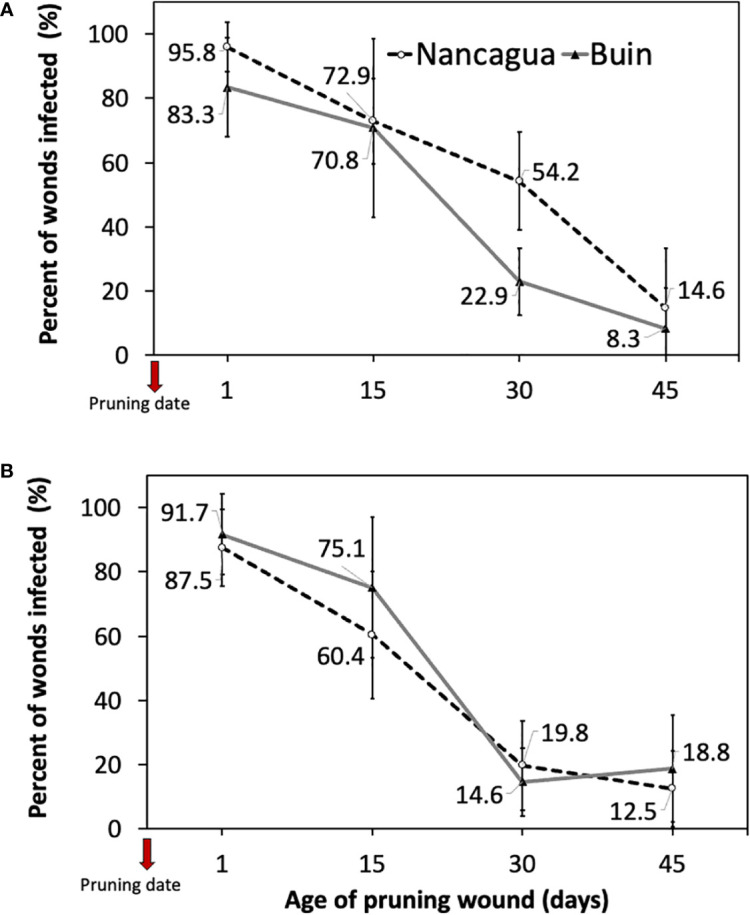
Percent of grapevine pruning wounds infected expressed as mean (%) ± standard deviation caused by artificial inoculation of 40 uL of a conidial suspension (10^5^ conidia/mL) of *Phaeomoniella chlamydospora* on pruning wound of different ages of spurs cv. Cabernet Sauvignon at 1, 15, 30, and 45 days after pruning in the commercial vineyards located in Buin (line) and Nancagua (segmented line), during eight months from the dormant season of 2014 **(A)** and 2015 **(B)** in Central Chile.

The analysis of the variance of the percentage of wounds infected showed an insignificant interaction between locality x age of pruning wounds (*P*=0.179; *F*=1.69) in 2014 and (*P*=0.397; *F*=1.07) in 2015 (**Table 3**). However, in 2014, the factor locality and age of pruning wounds had a significant effect of *P*=0.011 (*F*=6.91) and *P*<0.001 (*F*=49.31) on the % infected pruning wounds, respectively (**Table 3**), where the age of pruning wounds showed a significant reduction in recovery of *P. chlamydospora* according to the increase of the age of pruning wound (**Figure 6**). The spurs with an age of pruning wounds of 1 and 15 days were significantly more susceptible to infection by *P. chlamydospora* than those with age pruning wounds of 30 and 45 days, showing values of 95.8 to 70.8% of recovery compared with 22.9% and 8.3% (**Table 3**; **Figure 6A**).

**Table 3:** Percent of pruning wound infected (%) by artificial inoculation of *Phaeomoniella chlamydospora* at different ages of pruning wounds (1, 15, 30, and 45 days after pruning) in commercial vineyards cv. Cabernet Sauvignon during eight months in two localities (Buin and Nancagua), Central Chile.

Age of pruning wound (days)	Percent of pruning wounds infected (%)					
	Locality, 2014^a^			Locality, 2015^a^		
	Buin	Nancagua	Mean	Buin^a^	Nancagua^a^	Mean
1	83.3 bc	95.8 c	89.6	91.7 b	87.5 b	89.6
15	70.8 bc	72.9 bc	71.9	75.1 b	60.4 b	67.8
30	22.9 a	54.2 b	38.6	14.6 a	19.8 a	17.2
45	8.3 a	14.6 a	11.5	18.8 a	12.5 a	15.7
Mean	46.3	59.4		50.1	45.1	
*ANOVA*	df	*P*	SED^b^	df	*P*	SED^b^
Age (A)	3	0.011	4.9	3	< 0.001	3.8
Locality (L)	1	< 0.001	3.5	1	0.180	2.7
A x L	3	0.179	7.0	3	0.397	5.4

^a^Means of percent of pruning wounds infected (%) followed by the different lower case letters in each row and column differ significantly according to Tukey´s pairwise multiple comparison test. ^b^SED: Standard error of the difference.

In 2015, only the factor age of the pruning wound affected the recovery (%) of *P. chlamydospora* from inoculated spurs. In both the localities of Buin and Nancagua, the pruning wounds aged 1 and 15 days were significantly more susceptible to infection of *P. chlamydospore*, with values of 91.7 to 75.1% and 87.5% to 60.4% for 1 and 15 days after pruning, for Buin and Nancagua, respectively, compared with pruning wounds infected at 30 and 45 days after pruning (**Table 1**; **Figure 6B**). The susceptibility of wounds on spurs generally declined as the age of the pruning wound increased (**Figure 6**).

### Weather data

**Figure 7:**
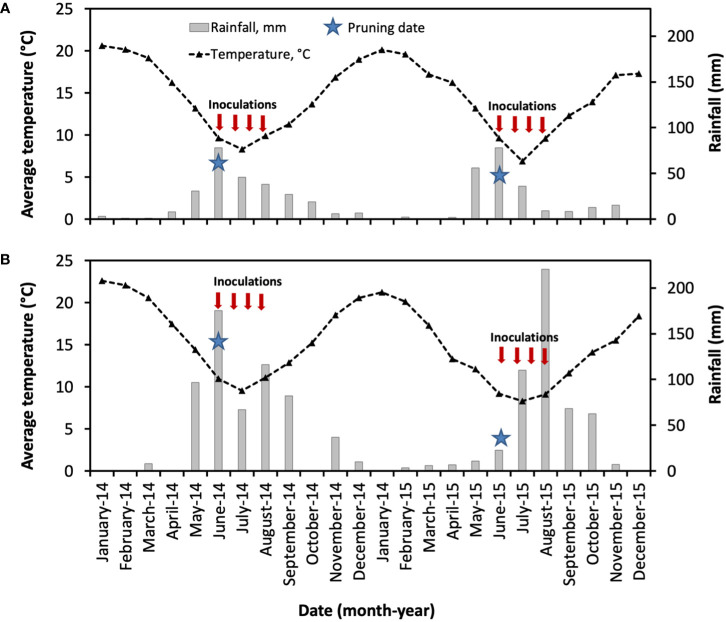
Weather conditions in the commercial vineyards cv. Cabernet Sauvignon showing the mean month of temperature (segmented line, °C) and month accumulated rainfall (gray bar, mm) located in Buin **(A)** and Nancagua **(B)** in Central Chile, during the seasons 2014 and 2015. Star indicates the date of pruning.

As shown in **Figure 7**, the weather conditions of the vineyards located in Central Chile were generally correspondent to the Mediterranean climate, with warmer and dry summer months from December to March. During this period, the average medium temperatures were 19°C (2014) and 18°C (2015) for Buin (Maipo Valley), and 22°C (2014) and 20°C (2015) for Nancagua (Cachapoal Valley). From May to August, the accumulated rainfall was 193 mm and 179 mm for Buin, and 455 mm and 364 mm for Nancagua in the seasons 2014 and 2015, respectively.

## Discussion

This study examined the susceptibility of different aged pruning wounds on grapevine cv. Cabernet Sauvignon to conidial inoculations of *P. chlamydospore* by evaluating rooted cuttings and spurs in two vineyards in Central Chile. Data obtained from two consecutive seasons of artificial inoculations showed that pruning wounds remained susceptible to *P. chlamydospora* for up 45 days after pruning. Natural infection was not detected in the non-inoculated controls. However, this pathogen was detected in very low prevalence at the sites of experimentation from previous work (Díaz and Latorre, 2014). This result was consistent with those obtained by Luque et al. (2014), where *P. chlamydospora* was isolated in only 12% of a diseased vineyard with over 20% prevalence of symptoms of GTDs compared with the healthy vineyard (0% of prevalence). The absence of *P. chlamydospora* from non-inoculated pruning wounds in the present work may possibly be due to their low prevalence in these vineyards, and to there being fewer opportunities for this low level inoculum to infect non-inoculated spurs in both vineyards. To determine the presence of conidia of *P. chlamydospora*, it is necessary to monitor these airborne spores using spore traps in the vineyards during the season and find the presence of pycnidia on the diseased vines (Edwards and Pascoe, 2001; Eskalen and Gubler, 2001; Eskalen et al., 2002; Eskalen et al., 2004). Moreover, studies performed in Australia by Edwards and Pascoe (2001), in California by Eskalen et al. (2002), and in South Africa by Baloyi et al. (2016) found pycnidia of *P. chlamydospora* on the surface of pruning debris left in the ground, as well as diseased cordons and old spurs. Epidemiologically, studies conducted in vineyards in California (Eskalen et al., 2002; Eskalen et al., 2004) and South Africa (Baloyi et al., 2016) have demonstrated that airborne conidia from pycnidia are viable and pathogenic. Therefore, pruning debris left in the ground, cordons, and old spurs attached to the grapevine are important inoculum sources in commercial vineyards (Díaz and Latorre, 2014; Baloyi et al., 2016; Gramaje et al., 2018).

In the Mediterranean weather conditions of Central Chile, wound susceptibility considerably decreased as the time between pruning and inoculation increased, reflecting the results of studies of *P. chlamydospora* carried out in vineyards in California (Eskalen et al., 2007), Italy (Serra et al., 2008), and South Africa (van Niekerk et al., 2011). In contrast, Elena and Luque (2016) found no seasonal variations in the susceptibility of pruning wound ages to *P. chlamydospora* in Spain. These differences may be attributed to differences in weather conditions and the grapevine cultivar evaluated. The temperature obtained in this study was not limiting for *P. chlamydospora*, because the range for mycelial growth (10 to 35°C) and germination (15 to 35°C) of *P. chlamydospora* is very wide (Whiting et al., 2001; Díaz and Latorre, 2014). In this sense, the monitored airborne conidia of *P. chlamydospora* have been correlated with the occurrence of rainfall or high relative humidity, especially during winter months in California (Eskalen et al., 2004), South Africa (van Niekerk et al., 2010), and Spain (González-Domínguez et al., 2020; Martínez-Diz et al., 2020). These weather parameters must therefore be studied in depth, specifically the factors involved in conidia dispersal, such as the rainfall and wind, and the distance reached by the airborne spore of *P. chlamydospora* in the vineyard. Furthermore, these aspects should be integrated along with the monitoring of airborne spores of *P. chlamydospora* during the seasons for a better understanding of the biology of this fungal trunk pathogen in Chilean commercial vineyards. Recently, González-Domínguez et al. (2020) developed a model for predicting periods with a high risk of *P. chlamydospora* airborne spore dispersal based on weather conditions. This model might be run using registered temperature and rainfall data together with airborne spore monitoring studies in different regions such as Central Chile to better understand *P. chlamydospora* biology and explain pruning wound susceptibility.

The results obtained in this study on Cabernet Sauvignon were in accordance with previous works conducted on other grapevine cultivars in California (Eskalen et al., 2007), Italy (Serra et al., 2008), and Spain (Elena and Luque, 2016), where a high percentage of *P. chlamydospora* was recovered from fresh pruning wounds in the vineyard during the first two weeks. Serra et al. (2008) found a decrease in susceptibility to *P. chlamydospora* during the growing season in Sauvignon Blanc, but differences in pruning dates were unclear. Eskalen et al. (2007) found that grapevine pruning wound susceptibility decreased over time in cvs. Thompson Seedless and Cabernet Sauvignon with a lower rate of susceptibility about 4 months after pruning, but the grapevine tissues remain susceptible to infection by *P. chlamydospora* from dormant to actively growing tissue. Although wound susceptibility to *P. chlamydospora* significantly decreased 45 days after artificial inoculation of pruning wounds, varying from 95.8 to 8.3% in 2014 and from 91.7 to 12.5% in 2015, the values of the percentage of wounds infected (95.8 to 60.4%) obtained from 1 to 15 days are epidemiologically important for the dissemination of *P. chlamydospora* in the vineyard over time (Serra et al., 2008; van Niekerk et al., 2011). These results do not discharge the possibility that root cuttings can be infected in the nursery process, as has been suggested previously in Chile (Díaz and Latorre, 2014). Furthermore, several studies have demonstrated the presence of fungal trunk pathogens, including *P. chlamydospora* on the propagation material of grapevines (Aroca et al., 2010; Gramaje and Armengol, 2011; Carbone et al., 2022).

The pruning date is considered to be a critical decision in the cultural practice and management of GTDs (Gramaje et al., 2018; Baumgartner et al., 2019). In this sense, several works have evaluated the effect of early versus late pruning on the level of infection of pruning wounds, where late pruning (February-March) can reduce the risk of infection to *P. chlamydospora*, because early pruning (December-January) coincides with the peak of spore release and the presence of the first rains of the season that generally occur in California (Eskalen and Gubler, 2001). Similarly, these results were also found with *E. lata* and *P. chlamydospora*, where early pruning inoculations showed higher levels of spurs infected in France (Chapuis et al., 1998) and Italy (Serra et al., 2008), respectively. This is in contrast to the results obtained by Luque et al. (2014) and Elena and Luque (2016), who obtained higher infection levels on spurs pruned late during the winter than early pruning in Spain. A similar result was obtained by van Niekerk et al. (2011); González-Domínguez et al. (2020), and Martínez-Diz et al. (2020), where later winter wounds were more susceptible to infection by *P. chlamydospora* than wounds made early in the dormant season in South Africa and Spain. Therefore, early and late pruning should be studied under Chilean conditions to know the effect of the pruning date on the infection by *P. chlamydospora* and other fungal trunk pathogens on grapevines.

Our results showed that fresh pruning wounds aged one and two weeks are more susceptible to artificial inoculation with *P. chlamydospora*. However, in the present work, the susceptibility was maintained for up to 45 days. Under these conditions, traditional protective management through a single fungicide application could not be enough in Chile (Díaz and Latorre, 2013). This same phenomenon was founded in the literature mentioned above, where the pruning wounds can be susceptible for a period extended up to 4 to 16 weeks after pruning in vineyards located in California (Petzoldt et al., 1981; Eskalen et al., 2007; Úrbez-Torres and Gubler, 2011), France (Chapuis et al., 1998), Italy (Serra et al., 2008), South Africa (van Niekerk et al., 2011), and Spain (Elena and Luque, 2016). Therefore, further studies evaluating one and two applications of fungicides and alternating with commercially available biocontrol agents are needed in Chile and worldwide.

## Data availability statement

The raw data supporting the conclusions of this article will be made available by the authors, without undue reservation.

## Author contributions

GD and BL contributed to the conceptualization and design of the study. GD conducted the experiments, formal analysis, and wrote the first draft of this manuscript. GD and BL contributed to manuscript revision, and read and approved the submitted version.

## Acknowledgments

The authors thank the reviewers for their critical comments on the manuscript. GD would like to send special thanks to Sra. Cecilia Ulloa for their valuable support and love (RIP).

## Conflict of interest

The authors declare that the study was conducted in the absence of any commercial or financial relationships that could be construed as a potential conflict of interest.

## Publisher’s note

All claims expressed in this article are solely those of the authors and do not necessarily represent those of their affiliated organizations, or those of the publisher, the editors and the reviewers. Any product that may be evaluated in this article, or claim that may be made by its manufacturer, is not guaranteed or endorsed by the publisher.
